# Enhancing Parental Well-being: Initial Efficacy of a 21-Day Online Self-help Mindfulness-Based Intervention for Parents

**DOI:** 10.1007/s12671-022-01998-1

**Published:** 2022-10-07

**Authors:** Rebecca Y. M. Cheung, Stanley K. C. Chan, Harold Chui, Wing Man Chan, Sammy Y. S. Ngai

**Affiliations:** 1 School of Psychology and Clinical Language Sciences, Harry Pitt Building, University of Reading, Earley Gate, Reading, RG6 6ES UK; 2 Department of Early Childhood Education, The Education University of Hong Kong, Hong Kong, Hong Kong; 3 New Life Psychiatric Rehabilitation Association, Hong Kong, Hong Kong; 4 Department of Educational Psychology, The Chinese University of Hong Kong, Hong Kong, Hong Kong

**Keywords:** Anxiety symptoms, Initial efficacy, Mindfulness-based intervention, Parents, Subjective well-being

## Abstract

**Objectives:**

Parental self-care is extremely important in the face of stress throughout parenthood. A 21-day online mindfulness-based intervention was developed that was aimed at enhancing parental well-being. The present study evaluated this intervention by examining its initial efficacy on parents’ mindfulness, parenting stress, subjective well-being, and symptoms of depression and anxiety.

**Methods:**

Participants were 273 parents (90.11% mothers) who were randomly assigned to the 21-day mindfulness-based intervention group (*n* = 136) or waitlist control group (*n* = 137). Pre-intervention assessment, immediate post-intervention assessment, and 30-day follow-up assessment were conducted to assess parents’ mindfulness, parenting stress, subjective well-being, and symptoms of depression and anxiety.

**Results:**

Linear mixed models indicated that the group × time effects on subjective well-being, anxiety symptoms, and mindfulness were significant, after controlling for sex, age, education, income, habit of mindfulness practice, hours of weekly mindfulness practice, and diagnostic history of psychiatric disorder. Follow-up analyses indicated that compared to baseline, participants from the intervention group reported significantly greater subjective well-being and mindfulness, and fewer symptoms of anxiety than did those from the waitlist control group. The group × time effects on parenting stress and depressive symptoms were non-significant. Exploratory findings further suggested practicality and perceived acceptability of the intervention.

**Conclusions:**

This study showed initial efficacy of a 21-day online mindfulness-based intervention on parents’ subjective well-being, anxiety symptoms, and mindfulness. The findings inform researchers and practitioners about the utility of a brief mindfulness-based intervention in promotion parental well-being. Other areas of feasibility warrant future investigation.

The study of mindfulness has flourished in the last few decades (Desrosiers et al., 2013; Garland et al., 2017a, b; Keng et al., 2011). Mindfulness is a mental state whereby people attend to the present-moment cognitive, emotional, and physical experiences without judgment (Kabat-Zinn, 1994). Mindfulness has been practiced for centuries in Buddhist and contemplative Christian communities to strengthen spirituality and religiosity (Kabat-Zinn, 2011; Trammel, 2015). Recent studies show that mindfulness is linked to better physical and psychological well-being, including fewer symptoms of depression and anxiety, lower perceived stress, reduced physical pain and impairment, better sleep, and better quality of life (Baer et al., 2012; Cheung & Ng, 2019; Finkelstein-Fox et al., 2019; Grossman et al., 2004). As a result, numerous secular mindfulness-based programs have emerged to enhance people’s well-being, regardless of their religious orientation, cultural tradition, profession, age, and clinical status (e.g., Benn et al., 2012; Bögels et al., 2010; Fendel et al., 2020; Kabat-Zinn, 2009; Segal et al., 2018).

Among existing mindfulness-based programs, the 8-week mindfulness-based stress reduction program (MBSR; Kabat-Zinn, 2009) is a group-intervention first developed in 1979 for chronically ill patients and has since shown mental health benefits across diverse populations, including adolescents and adults, in both clinical and non-clinical settings (e.g., Chi et al., 2018; Grossman et al., 2004). With reference to the MBSR and mindfulness-based cognitive therapy (MBCT; Segal et al., 2018), another 8-week program on mindful parenting (Bögels & Restifo, 2013; Bögels et al., 2010) and its modified versions (e.g., Boekhorst et al., 2021) have also received much attention, demonstrating benefits beyond parenting practices, including better parents’ and children’s mental health and adjustment, better coparenting practices, better self-compassion among parents, and reduced parenting stress (Boekhorst et al., 2021; Bögels et al., 2014; Emerson et al., 2021; Ma & Siu, 2016; van der Oord et al., 2012). Although mindfulness-based interventions have been developed to enhance parents’ well-being, especially for parents with a child with special needs (e.g., Bazzano et al., 2015; Benn et al., 2012; Lunsky et al., 2021), most of the programs are either family-focused (e.g., Coatsworth et al., 2015) or parenting-focused (Bögels & Restifo, 2013; Ferraioli & Harris, 2013; Petcharat & Liehr, 2021; Shaffer et al., 2020), with few focusing primarily on parental well-being. Indeed, one of the core aspects of mindful parenting programs is to foster parental well-being (e.g., Bögels et al., 2010), as parents who are well are also more likely to respond skillfully to the child (e.g., Cheung et al., 2019; Jones et al., 2014; Miner, 2019). Nevertheless, well-being programs are also important, as they offer practical strategies for parents to tackle with well-being challenges during parenthood (Camisasca et al., 2016; Johansson et al., 2020).

According to mindfulness-to-meaning theory (Garland et al., 2015), mindfulness is associated with well-being via mindful emotion regulation. Mindfulness facilitates decentering from negative stress appraisal to metacognitive awareness. For instance, parents who are mindful may disengage themselves from autopilot responses to stress (e.g., reacting negatively to a spouse/a child upon the arising of competing demands from an employer). Instead, they may pause, decenter from immediate reactions, and broaden their awareness to previously unattended details (e.g., availability of alternative solutions to reduce work-family conflict). The shift of attention and awareness further increases people’s ability to regulate emotions and reappraise negative experiences to a positive light (Teper et al., 2013). Recent findings based on mindfulness-to-meaning theory show that mindfulness fosters adaptive emotion regulation, such as positive reappraisal and savoring, thereby increasing positive emotions and mental well-being (e.g., Bryant & Smith, 2015; Cheung & Lau, 2021; Cheung & Ng, 2020; Garland et al., 2017a, b, 2021; McConnell & Froeliger, 2015). Similar findings are also indicated among parents, in that mindfulness or mindful parenting behavior is linked to lower parenting stress and better mental health (e.g., Burgdorf et al., 2019; Cheung et al., 2019; Moreira & Canavarro, 2018).

Consistent with mindfulness-to-meaning theory (Garland et al., 2015), parents who took part in mindfulness-based programs experienced better mental health and lower perceived stress (e.g., Bazzano et al., 2015; Benn et al., 2012). For instance, parents of children with developmental disabilities who completed an 8-week MBSR program experienced greater mindfulness, psychological well-being, and self-compassion, as well as lower general and parental stress (Bazzano et al., 2015). Similarly, upon completion of a 5-week mindfulness training, parents and educators of children with special needs experienced greater mindfulness, self-compassion, and personal growth than did people from the waitlist control group (Benn et al., 2012). Furthermore, parents of children with autism who took part in a 6-week group virtual MBCT reported greater mindfulness, lower stress, and fewer depressive symptoms (Lunsky et al., 2021). Although the above studies involved caregivers of children with disabilities or special needs, recent findings indicated that the positive effects of mindful parenting training did not differ between families from clinical and non-clinical settings (Potharst et al., 2021). Hence, mindfulness training is beneficial to parents of children with and without special needs.

Given the mental health benefits of mindfulness, online mindfulness-based and mindfulness-related interventions for parents have burgeoned recently (e.g., Boekhorst et al., 2021; Flynn et al., 2020; Potharst et al., 2019; Shaffer et al., 2020; see Sommers-Spijkerman et al., 2021, for a meta-analytic review of the effects of online mindfulness-based interventions on mental health). The online format offers parents the appeals of flexibility (e.g., reduced costs and efforts of transportation, options to go over recordings after class and to attend to family matters during break time), especially in times of the COVID-19 pandemic (Lunsky et al., 2021). While some of these interventions are conducted in real-time settings (Lunsky et al., 2021), others are asynchronous (Aller et al., 2020; Boekhorst et al., 2021), including the intervention developed in this study. As parents often face caregiving and other responsibilities such as household chores, work-family balance, and financial demands (Hook et al., 2022; Ponnet et al., 2016; Ruppanner et al., 2021), short-term asynchronous interventions allow flexibility for parents to participate at their convenience and reduce the burdens of scheduling. Once an asynchronous program has been developed, the cost of administration is also much lower than synchronous, real-time interventions, most of which require the presence of a qualified or certified mindfulness teacher. Hence, online asynchronous mindfulness-based interventions are preferred by adults (Wahbeh et al., 2014) and show preliminary evidence in improving attention and reducing stress and anxiety among nursing students (Spadaro & Hunker, 2016). With peer support, online asynchronous mindfulness-based training for parents and family carers of people with intellectual disabilities has also demonstrated acceptability and feasibility (Flynn et al., 2020). Despite the advantages, the disadvantages of asynchronous MBIs should also be noted, including the removal of a group context and a heavy reliance on self-learning, in addition to the absence of teacher-guided inquiries, peer interactions, and a responsive teacher (Cavanagh et al., 2014).

While numerous mindfulness-based programs for parents’ well-being have been evaluated in the Western context (Bazzano et al., 2015; Benn et al., 2012; Lunsky et al., 2021), few have been developed and examined in Asian contexts, such as Hong Kong. As a densely populated city involving fast-paced living and a soaring housing market (Lam, 2021), parents not only face parental stress as caregivers, but also other types of demands, such as household crowdedness and economic stress. Regarding work-family balance, a recent government report shows that local men and women had a median of 44.4 and 42.6 weekly working hours, respectively (Census and Statistics Department, Hong Kong Special Administrative Region, 2021b), totaling 2088 annual working hours. This figure was greater than the worldwide average annual working hours (Organisation for Economic Co-operation and Development, n.d.) and indicated potential job stress and burnout (e.g., Ng et al., 2020; Tsang, 2018). In addition, compared to (non-Asian) American parents, Chinese parents tend to provide much more direct (e.g., offering homework help) and indirect (e.g., giving children fewer household chores) assistance to support children’s learning (Ng & Wei, 2020). These practices increase parental involvement and potential burden among the parents. As such, the stress and demands encountered by parents in Hong Kong may undermine their overall mental health (Lam, 2015; Wong et al., 2003).

Based on Bowen et al.’s (2009) conceptualization of feasibility studies, this study aimed to evaluate a core aspect of feasibility, i.e., initial efficacy, of a self-help online asynchronous mindfulness intervention for parents entitled “New Life Mindfulness-based Program for Parents” (NL-MBP). The study also explored the practicality and the perceived acceptability of NL-MBP, as reflected by participants’ frequency of mindfulness practice over a 21-day period, their knowledge and attitudes about mindfulness and self-care, their perceived usefulness of NL-MBP, and whether they would recommend NL-MBP at post-intervention. It was hypothesized that compared to parents in the waitlist control group, those enrolled in the 21-day mindfulness-based intervention would have greater mindfulness, lower parental stress, and better mental health, as indexed by greater subjective well-being and fewer symptoms of depression and anxiety at the post-intervention assessment, after controlling the effects of sex, age, education, income, habit of mindfulness practice, hours of weekly mindfulness practice, and diagnostic history of psychiatric disorder. Similarly, it was hypothesized that the benefits of the intervention would be sustained at the 30-day follow-up.

## Method

### Participants

**Fig. 1:**
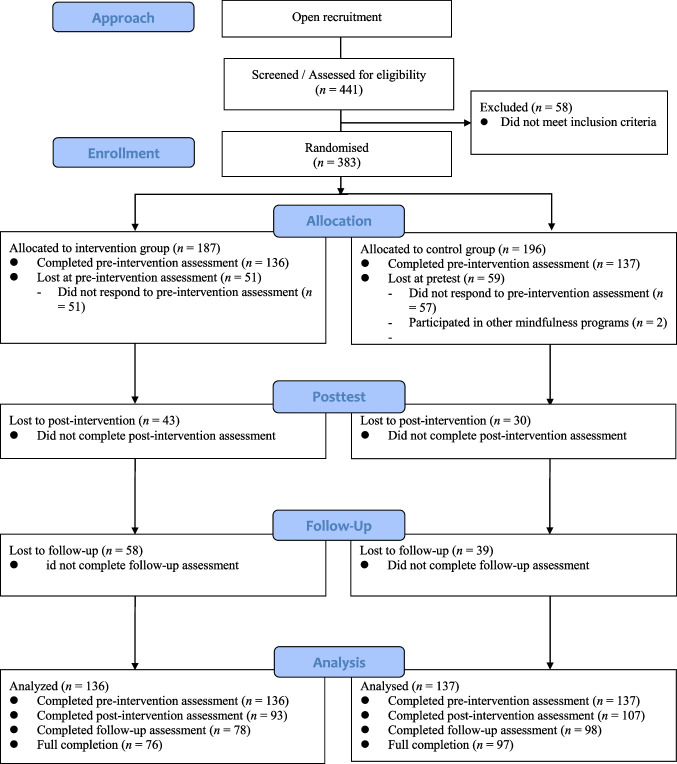
CONSORT flowchart for participants: recruitment and retention

A total of 441 Chinese parents were recruited from various sources, including parent-teacher associations in local primary schools, social media, and one-off mindfulness workshops organized for parents in primary schools, who went through a screening process. The inclusion criteria were as follows: (a) being proficient in Chinese, (b) having a child at a local primary school, (c) not being at risk for self-harm, and (d) did not have a relapse of psychiatric disorder in the past 6 months. Among them, 58 parents did not meet the inclusion criteria. Therefore, 383 eligible parents were randomized to the intervention group (*n* = 187) or waitlist control group (*n* = 196). Figure 1 shows the CONSORT flow diagram of recruitment and retention. Among the 383 participants, 110 were excluded because they either did not complete the pre-intervention assessment (*n* = 108) or were participating in other mindfulness-based interventions at that moment (*n* = 2). The final sample included 136 parents for the intervention group and 137 parents for the waitlist control group. Table 1 presents the demographic information of the participants. Chi-square tests and independent samples *t*-tests indicated that participants from the intervention group and the waitlist control group did not differ in the demographic variables and the core study variables (*p*s > 0.05), except for depressive symptoms (*t*(271), − 2.33, *p* = 0.02, Cohen’s *d* = 0.08), with those from the intervention group reporting a lower level of depressive symptoms (*M* = 7.51, *SD* = 4.55) than those from the control group (*M* = 8.92, *SD* = 5.43).

**Table 1:** Demographic information of participants

	Intervention group (*n* = 136) percentage/mean (SD)	Waitlist control group (*n* = 137) percentage/mean (SD)	Group difference *p* values (two-tailed)
Gender			.15
Male	12.50%	7.30%	
Female	87.50%	92.70%	
Age (in years)	41.37 (5.83)	41.58 (4.89)	.51
Monthly family income (in Hong Kong Dollar)			.08
< $10,000	8.09%	4.38%	
$10,001–$30,000	23.53%	19.71%	
$30,001–$50,000	29.41%	25.55%	
$50,001–$70,000	20.58%	10.95%	
$70,001–$90,000	5.88%	12.41%	
> $90,001	10.29%	16.79%	
Unknown/Not available	4.41%	7.30%	
Educational level			.60
Primary education or below	0.74%	0.73%	
Form 1–3 of secondary education	2.94%	2.19%	
Form 4–7 of secondary education	18.38%	13.14%	
Associate/Diploma/Undergraduate education	51.47%	57.66%	
Graduate degree or above	26.47%	25.55%	
Unknown/Not available	0.00%	0.73%	
Marital status			.54
Single	0.74%	0.73%	
Married	92.65%	94.16%	
Divorced	5.15%	4.38%	
Living separately	0.00%	0.73%	
Widowed	1.47%	0.00%	
Knowledge about mindfulness			.09
Unfamiliar	41.18%	34.31%	
A bit	54.41%	64.23%	
Quite familiar	4.41%	0.73%	
Very familiar	0.00%	0.73%	
Habit of practicing mindfulness			.88
Yes	13.24%	13.87%	
No	86.76%	86.13%	
Mindfulness activities with children			.89
Yes	16.18%	16.79%	
No	83.82%	83.21%	

The attrition rate of the present study was comparable to other studies involving self-help interventions (e.g., 27%; Cavanagh et al., 2014) or internet-based treatments (e.g., 31%; Melville et al., 2010). Specifically, within the intervention group (*n* = 136), the attrition rate was 31.62% (*n* = 43) at post-intervention and 16.13% (*n* = 15) at 30-day follow-up. No significant differences were found between dropouts and post-intervention completers for all variables under study (*p*s > 0.05). However, at follow-up, the dropouts and the retained participants differed in age (*t*(134) =  − 2.12, *p* = 0.36) and mindfulness (*t*(94) =  − 2.95, *p* = 0.004). Participants who completed the follow-up were significantly older (*M* = 42.27, *SD* = 6.27) and more mindful (*M* = 65.34, *SD* = 7.16) than were the dropouts (age *M* = 40.16, *SD* = 4.98; mindfulness *M* = 59.70, *SD* = 9.20). Within the waitlist control group (*n* = 137), the attrition was 21.90% (*n* = 30) at post-intervention and 8.41% (*n* = 9) at the 30-day follow-up. No significant differences were found between completers and dropouts at either time point for all variables (*p*s > 0.05). Little’s missing completely at random test was conducted to test the null hypothesis of data missing completely at random. The finding was non-significant (*χ*^2^(198) = 173.47, *p* = 0.90), suggesting the data were missing completely at random.

### Procedures

Following the group allocation, participants completed the pre-intervention online questionnaires via REDCap electronic data capture tools (Harris et al., 2009, 2019). Although the online self-help intervention was not originally designed in response to the COVID-19 pandemic, it was conducted between May 2020 and January 2021 during the fluctuating pandemic. For the intervention group, participants received the 21-day mindfulness-based program instructions upon their completion of the pre-intervention assessment at baseline. Specifically, a set of guided mindfulness practice recording and its corresponding reading was sent to participants daily via WhatsApp, a mobile instant messaging application commonly used in Hong Kong. Participants completed the practice on their mobile phones asynchronously at their convenience, regardless of the location. After 21 days, participants received the post-intervention questionnaire via REDCap. A WhatsApp reminder was sent to participants if they had not responded to the questionnaire within 3 days. Upon completion of the post-intervention assessment, a follow-up assessment was delivered to the participants via REDCap after 30 days. Participants received a supermarket coupon at HK$40 (~ US$5) for their time and effort upon completion of the pre-intervention and post-intervention assessments. To minimize attrition, participants who completed the pre-intervention, post-intervention, and follow-up assessments received an additional supermarket coupon at HK$60 (~ US$7.5).

As for the waitlist control group, similar procedures were conducted at baseline, post-intervention, and follow-up. Participants in the waitlist control group also received the same incentives described above. Upon completion of the follow-up assessment, they received the 21-day guided mindfulness practice recordings and the corresponding readings.

#### The 21-Day Self-help Mindfulness-Based Intervention

The self-help online mindfulness-based intervention for parents, entitled “New Life Mindfulness-based Program for Parents” (NL-MBP), is an asynchronous intervention based on MBSR (Kabat-Zinn, 2009) and MBCT (Williams & Penman, 2011). The intervention was designed by a registered educational psychologist, who was also an experienced mindfulness teacher in Hong Kong. The 21-day intervention aimed to increase mindfulness through eight-core mindfulness practices, namely mindful eating, body scan, mindful breathing, mindful stretching, mindful walking, self-care exercise, breathing space, and lovingkindness practice. To promote their understanding and application of mindfulness, parents were invited to engage in a daily 10- to 15-min guided mindfulness practice and a corresponding one-page reading for 21 consecutive days (see Table 2). Previous research indicated that 21 days of mindful breathing practices could lead to an overall change in habit automaticity (Lewis et al., 2021; see also Maltz, 1969) and health outcomes (Li et al., 2022). Based on the evidence, 21 practices and corresponding readings were developed for the intervention. In the present study, each set of the recording and reading was presented to the participants simultaneously every day. Participants were encouraged daily to complete the practices at their convenience. The content of NL-MBP included (a) general information on mindfulness, such as its misconceptions and potential benefits, (b) description of the mindfulness practices, (c) skillful responses to encounter common difficulties, especially in the family setting, (d) seven attitudes in cultivating mindfulness (i.e., non-judging, patience, beginner’s mind, trust, non-striving, acceptance, and letting go; Kabat-Zinn, 2012), and (e) application of mindfulness in daily lives. Although the 21-day intervention offered parents mindfulness exercises for self, family scenarios were included to enhance their interest and motivation to practice as a parent or a family member. For example, one of the mindful breathing recordings included the following excerpt, “have you ever noticed your breathing? When you are chasing the school bus with your child, your breathing is faster than usual. When you are drawing a picture with your child, your breathing is more stable and slower. Let’s practice the awareness of our breath together…” It should be noted that the NL-MBP was not a family-focused intervention for family members to practice mindfulness together. With aims to promote parental well-being, the NL-MBP also was not a mindful parenting intervention.

**Table 2:** Themes of the 21-day self-help mindfulness-based intervention

Day	Recording	Reading
1	Mindful eating	Introduction to mindfulness
2	Body scan (1)	How to practice a body scan?
3	Body scan (2)	Possible situations during body scanning
4	Body scan (3)	Benefits of mindfulness
5	Body scan (4)	Emphasizing on non-judging and patience
6	Mindful breathing (1)	Introduction to mindful breathing
7	Mindful breathing (2)	Welcoming distractions in mindfulness practice
8	Mindful breathing (3)	Misconceptions of mindfulness (1)
9	Mindful breathing (4)	Emphasizing on beginner’s mind and trust
10	Mindful stretching (1)	Introduction to mindful stretching
11	Mindful stretching (2)	Exploring and sensing your limits
12	Mindful stretching (3)	Knowing your dark side and living in the moment
13	Mindful stretching (4)	Emphasizing on non-striving and acceptance
14	Mindful walking (1)	Introduction to mindful walking
15	Mindful walking (2)	Misconceptions of mindfulness (2)
16	Self-compassion exercise (1)	Introduction to self-compassion
17	Self-compassion exercise (2)	Self-compassion for parents
18	Self-compassion exercise (3)	Emphasizing on letting go
19	“Take a step back, give yourself a breather” (1)	Listening attentively to your own feelings
20	“Take a step back, give yourself a breather” (2)	Importance of taking a step back
21	Mindful breathing and self-reflection	Conclusion

### Measures

#### Subjective Well-being

The five-item World Health Organization Well-being Index (WHO-5; World Health Organization, 1998) was used to measure parents’ subjective well-being. All items were rated on a 6-point scale ranging from 0 (*never*) to 5 (*all the time*). Sample items included “I have felt cheerful and in good spirits” and “I have felt active and vigorous.” An overall score was calculated by summing the item scores, with higher scores indicating a better quality of life. Previous studies reported good internal consistency, convergent validity, and sensitivity to change of the scale (e.g., Newnham et al., 2010). In this study, Cronbach’s alpha = 0.91 and McDonald’s *ω* = 0.91.

#### Anxiety Symptoms

The seven-item Generalized Anxiety Disorder Scale (GAD-7; Spitzer et al., 2006) was used to measure anxiety symptoms on a 4-point scale, ranging from 0 (*not at all*) to 3 (*nearly every day*). The item scores were summed to form a total score, with higher scores indicating greater anxiety. Sample items included “Feeling nervous, anxious or on edge” and “Not being able to stop or control worrying.” The Chinese version of GAD-7 was previously validated in Chinese individuals (e.g., He et al., 2010). In this study, Cronbach’s alpha = 0.92 and McDonald’s *ω* = 0.92.

#### Depressive Symptoms

The 9-item Patient Health Questionnaire (PHQ-9; Kroenke et al., 2001) was used to assess symptoms of depression. Participants described how often they experienced any depressive symptoms in the past 2 weeks. Items were rated on a 4-point scale from 0 (*not at all*) to 3 (*almost every day*). A sample item is as follows, “In the past two weeks, I had less interest and fun in doing activities.” A total score was calculated by summing the item scores, with higher scores indicating greater severity of depression. The Chinese version of PHQ-9 was validated in the Chinese context and indicated good validity and internal consistency (e.g., Yu et al., 2012). In this study, Cronbach’s alpha = 0.87 and McDonald’s *ω* = 0.87.

#### Mindfulness

The 20-item Five Facet Mindfulness Questionnaire – Short Form (FFMQ-SF; Hou et al., 2014) was used to assess mindfulness on five facets, namely observing, describing, acting with awareness, non-reactivity, and non-judging (Baer et al., 2008). Participants rated on a 5-point scale, ranging from 1 (*never*) to 5 (*always*). Sample items included “I pay attention to sensations, such as the wind in my hair or sun on my face” and “I’m good at finding words to describe my feelings.” A total score was computed by summing the item scores, with higher scores indicating greater mindfulness. In this study, Cronbach’s alpha = 0.80 and McDonald’s *ω* = 0.73.

#### Parental Stress

The 17-item Parental Stress Scale (PSS; Berry & Jones, 1995) was used to measure parents’ subjective feelings of difficulties, dissatisfaction, and strains. All items were rated on a 5-point scale from 1 (*strongly disagree*) to 5 (*strongly agree*). Sample items included “I am happy in my role as a parent” and “Caring for my children sometimes takes more time and energy than I have to give.” A total score was obtained by summing the item scores, with higher scores indicating greater stress. The Chinese version of the PSS was previously validated and showed high internal consistency (Cheung, 2000). In this study, Cronbach’s alpha = 0.70 and McDonald’s *ω* = 0.89.

### Data Analyses

Tests of descriptive statistics were conducted to explore participants’ frequency of practice and perceived acceptability. Linear mixed models (LMMs) were conducted via the lme4 package (Bates et al., 2014) in R version 4.1.1 (R Core Team, 2021) to examine between-group differences (intervention vs. waitlist control group) over time (pre-intervention, post-intervention, and follow-up), after controlling the effects of sex, age, education, income, habit and hours of weekly mindfulness practice, and diagnostic history of psychiatric disorder, with maximum likelihood as the estimation method. Given our attrition of 31.62% and 16.13% at post-intervention and follow-up, respectively, LMM was conducted via the missing at random assumption. By estimating random subject effects, participants with missing data were included, with observed values being used to detect the pre-post changes between groups. The *p* values for fixed effects coefficients were acquired using Satterthwaite’s method of approximations of degrees of freedom. Upon detection of significant group × time effects, follow-up within- and between-group analyses were conducted using *t*-tests.

## Results

### Frequency of Practice

A total of 43 participants from the intervention group recorded their frequency of practice that indicated their degree of program completion. Specifically, 25.58% and 48.84% reported they had completed the guided meditations and readings every day, respectively, whereas 46.51% and 16.28% completed 15 to 20 days of guided meditations and readings, respectively. In addition, 9.30% and 16.28% reported they had completed 9 to 14 days of guided meditations and readings, respectively, whereas 13.95% and 11.63% completed 3 to 8 days of guided meditations and readings, respectively. Finally, 4.65% and 6.98% of the participants reported that they had completed fewer than 3 days of guided meditations and readings, respectively. Participants who recorded their frequency of practice did not differ from the others in the intervention group on all study variables, including mindfulness, subjective well-being, symptoms of depression and anxiety, sex, age, education, income, habit of mindfulness practice, hours of weekly mindfulness practice, and diagnostic history of psychiatric disorder (*p*s > 0.05). However, participants who recorded their frequency of practice reported higher parental stress (*M* = 55.79, *SD* = 9.48) than those who did not (*M* = 49.90, *SD* = 12.13) (*t*(93) =  − 2.59, *p* = 0.011). Follow-up analyses indicated that a greater frequency of mediation practice was associated with better subjective well-being at baseline (*r* = 0.31, *p* = 0.043) and post-intervention (*r* = 0.50, *p* = 0.001). A greater frequency of mediation practice was also associated with fewer depressive symptoms (*r* =  − 0.32, *p* = 0.39) and greater mindfulness at post-intervention (*r* = 0.46, *p* = 0.002), but not with the other variables or time points (*p*s > 0.05). Similarly, greater completion of readings was associated with greater subjective well-being (*r* = 0.41, *p* = 0.006), fewer depressive symptoms (*r* =  − 0.33, *p* = 0.03), and greater mindfulness at post-intervention (*r* = 0.47, *p* = 0.002). It was also associated with greater mindfulness at baseline (*r* = 0.40, *p* = 0.008) and follow-up (*r* = 0.47, *p* = 0.01), but not with the other variables or time points (*p*s > 0.05).

### Perceived Acceptability

A total of 44 participants from the intervention group rated their perceived acceptability of the intervention (Bowen et al., 2009). Notably, all participants agreed the intervention was useful, 97.73% reported that the intervention increased their knowledge about mindfulness, 95.45% reported that the intervention helped them realize the importance of self-care, 86.36% noted that they would recommend the intervention to others, and 81.82% expressed they would continue practicing mindfulness.

### Group × Time Effects

At baseline, participants did not differ between groups on subjective well-being (*M*_intervention_ = 8.93, *SD* = 4.21; *M*_control_ = 8.58, *SD* = 4.56), anxiety (*M*_intervention_ = 7.81, *SD* = 4.60; *M*_control_ = 8.82, *SD* = 5.45), depressive symptoms (*M*_intervention_ = 7.51, *SD* = 4.55; *M*_control_ = 8.92, *SD* = 5.43), mindfulness (*M*_intervention_ = 59.87, *SD* = 7.84; *M*_control_ = 59.28, *SD* = 7.94), and parental stress (*M*_intervention_ = 55.76, *SD* = 11.54; *M*_control_ = 57.39, *SD* = 12.48) (*p*s > 0.05). Similarly, participants in full completion of the study did not differ between groups in subjective well-being (*M*_intervention_ = 9.27, *SD* = 4.24; *M*_control_ = 8.67, *SD* = 4.87), anxiety (*M*_intervention_ = 7.29, *SD* = 4.51; *M*_control_ = 8.29, *SD* = 5.13), depressive symptoms (*M*_intervention_ = 7.06, *SD* = 4.58; *M*_control_ = 8.37, *SD* = 5.28), mindfulness (*M*_intervention_ = 60.23, *SD* = 7.28; *M*_control_ = 60.19, *SD* = 7.65), and parental stress (*M*_intervention_ = 55.62, *SD* = 11.59; *M*_control_ = 57.37, *SD* = 12.80) (*p*s > 0.05).

**Fig. 2:**
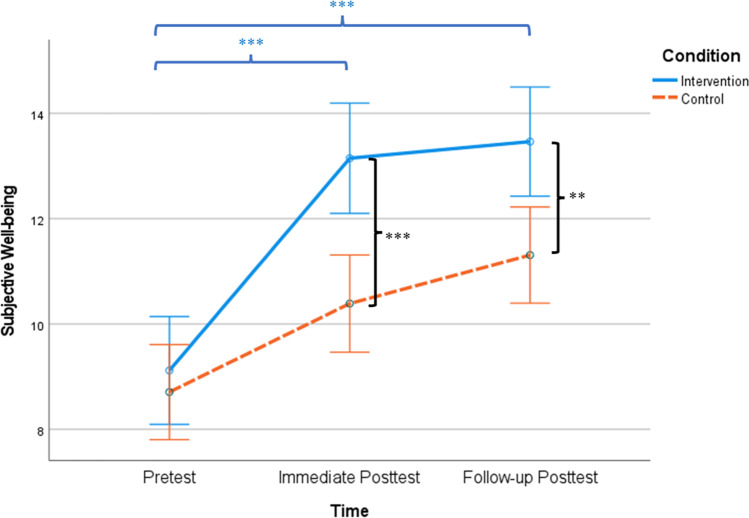
Subjective well-being between conditions over time. *Note.* ^*^p < .05; ^**^p < .01; ^***^p < .001. Asterisks between the solid and dashed lines denote between-group differences at each time point. Asterisks on the top denote significant differences between baseline, post-intervention, and follow-up within the intervention group

LMM revealed significant group × time effect on subjective well-being (*F*(1, 436.18) = 16.75, *p* < 0.001, CI: − 1.72, − 0.60, ${\eta }_{p}^{2}$= 0.04), after controlling for of age, sex, education, income, habit and hours of weekly mindfulness practice, and diagnostic history of psychiatric disorder. Follow-up analyses suggested that compared to baseline (*M*_intervention_ = 8.93, *SD* = 4.21; *M*_control_ = 8.58, *SD* = 4.56), participants from the intervention group reported significantly greater subjective well-being than did those from the control group at post-intervention (*t*(207) = 4.78, *p* < 0.001, Cohen’s *d* = 0.66; *M*_intervention_ = 13.18, *SD* = 4.62; *M*_control_ = 10.09, *SD* = 4.71) and at follow-up (*t*(178) = 3.30, *p* = 0.001, Cohen’s *d* = 0.50; *M*_intervention_ = 13.56, *SD* = 4.68; *M*_control_ = 11.24, *SD* = 4.67). Within-group analyses indicated that compared to baseline, participants from the intervention group had a significant increase of subjective well-being at post-intervention (*t*(97) =  − 9.78, *p* < 0.001, Cohen’s *d* =  − 0.99) and at follow-up *(t*(78) =  − 8.79, *p* < 0.001, Cohen’s *d* =  − 0.99). Participants from the control group also had a significant and smaller increase of subjective well-being at post-intervention (*t*(110) =  − 4.39, *p* < 0.001, Cohen’s *d* =  − 0.42) and at follow-up (*t*(100) =  − 6.70, *p* < 0.001, Cohen’s *d* =  − 0.67). See Fig. 2 for details.

**Fig. 3:**
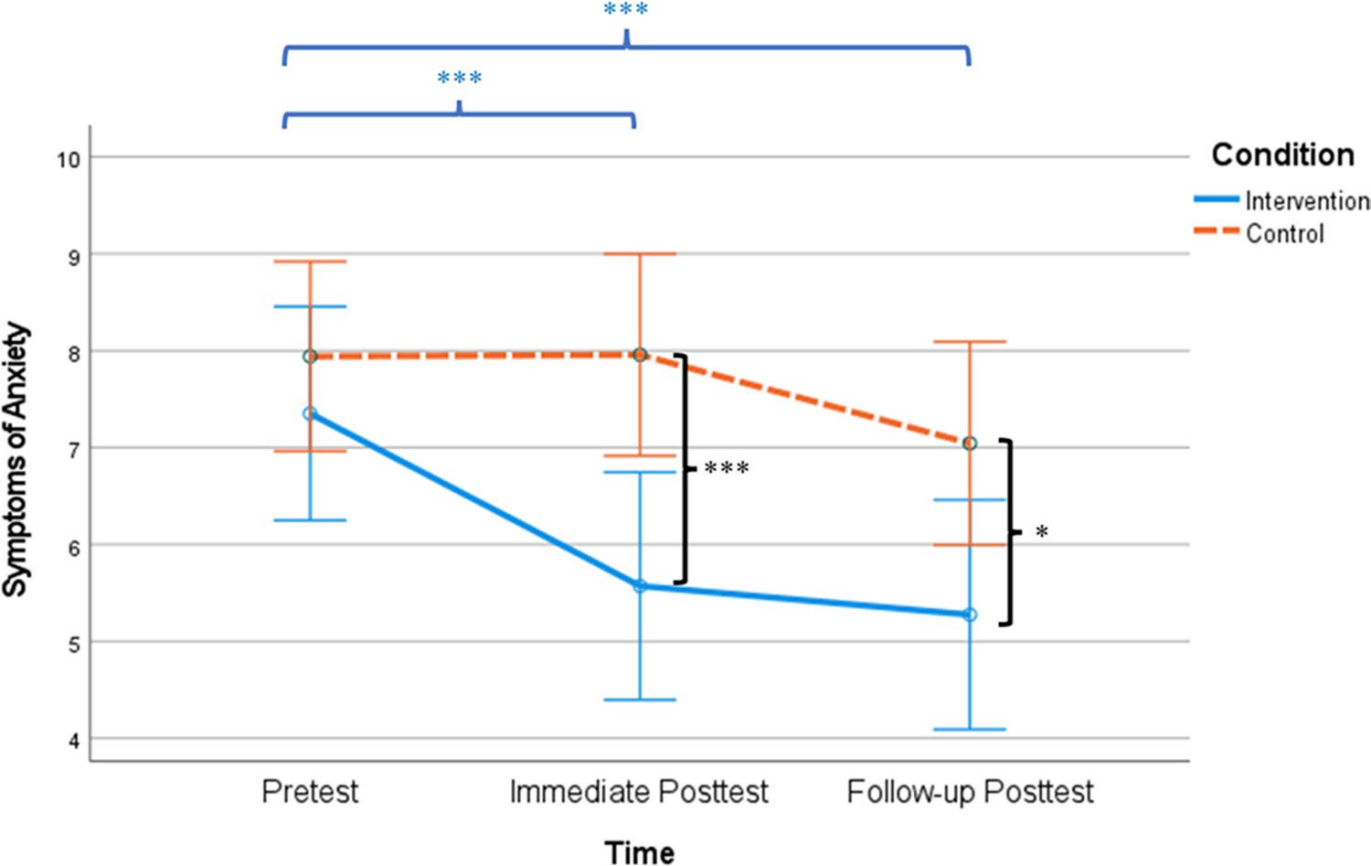
Symptoms of anxiety between conditions over time. *Note.* ^*^p < .05; ^**^p < .01; ^***^p < .001. Asterisks between the solid and dashed lines denote between-group differences at each time point. Asterisks on the top denote significant differences between baseline, post-intervention, and follow-up within the intervention group

LMM also revealed significant group × time effect on anxiety (*F*(1, 421.47) = 4.35, *p* = 0.04, CI: 0.04, 1.38, ${\eta }_{p}^{2}$= 0.01), after controlling for age, sex, education, income, habit and hours of weekly mindfulness practice, and diagnostic history of psychiatric disorder. Follow-up analyses suggested that compared to baseline (*M*_intervention_ = 7.81, *SD* = 4.60; *M*_control_ = 8.82, *SD* = 5.45), participants from the intervention group reported significantly lower anxiety levels than did those from the control group at post-intervention (*t*(201) =  − 4.06, *p* < 0.001, Cohen’s *d* =  − 0.57; *M*_intervention_ = 5.50, *SD* = 4.73; *M*_control_ = 8.50, *SD* = 5.70) and at follow-up (*t*(171) =  − 2.56, *p* = 0.01, Cohen’s *d* =  − 0.39; *M*_intervention_ = 5.12, *SD* = 4.79; *M*_control_ = 7.25, *SD* = 5.92). Within-group analyses indicated that compared to baseline, participants from the intervention group had a significant reduction of anxiety symptoms at post-intervention (*t*(95) = 4.15, *p* < 0.001, Cohen’s *d* = 0.42) and at follow-up (*t*(76), 3.96, *p* < 0.001, Cohen’s *d* = 0.45). In comparison, participants from the control group did not have significant reductions of anxiety at post-intervention and follow-up (*p*s > 0.05). See Fig. 3 for details.

**Fig. 4:**
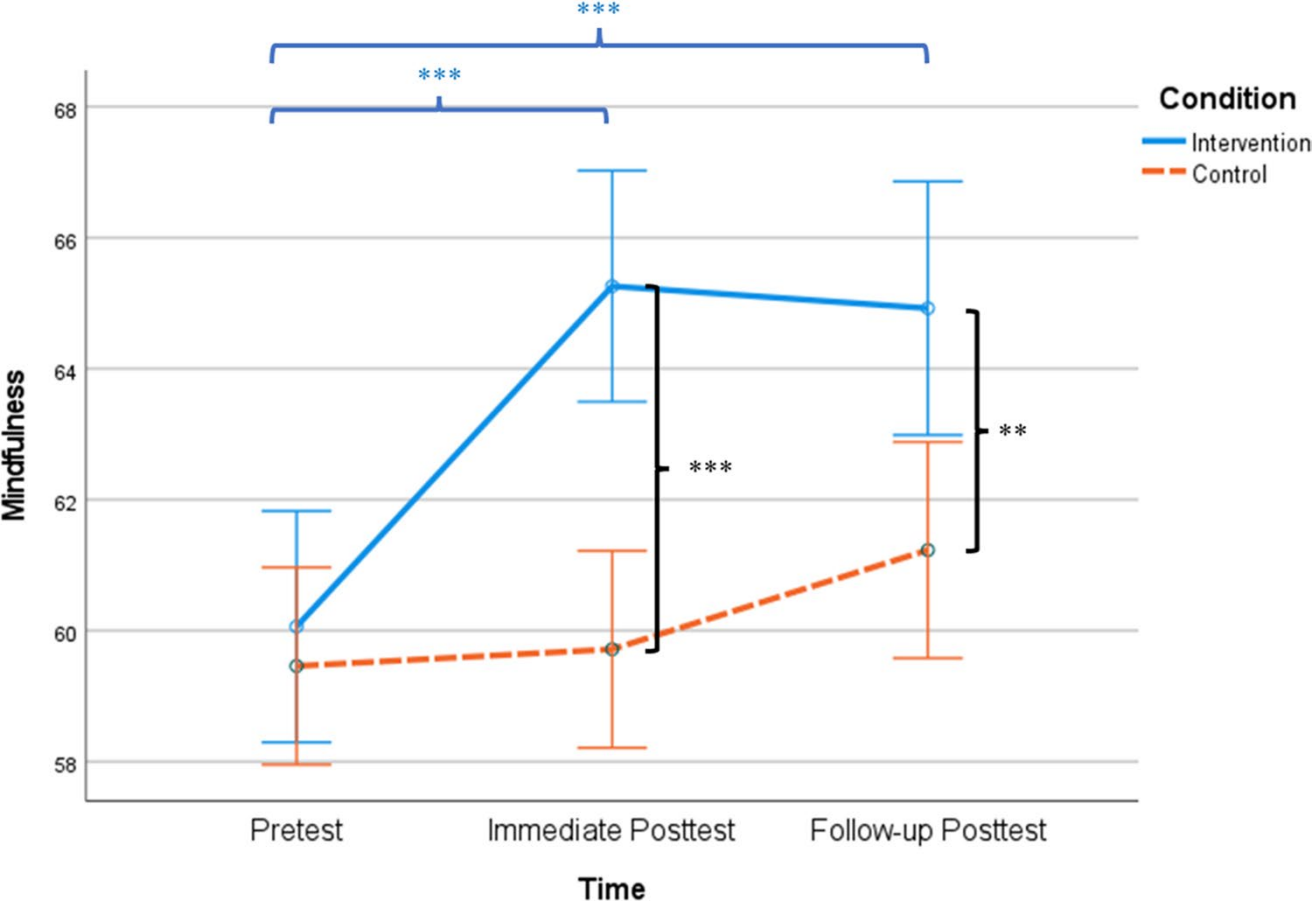
Mindfulness between conditions over time. *Note.* ^*^p < .05; ^**^p < .01; ^***^p < .001. Asterisks between the solid and dashed lines denote between-group differences at each time point. Asterisks on the top denote significant differences between baseline, post-intervention, and follow-up within the intervention group

Next, LMM revealed significant group × time effect on mindfulness (*F*(1, 409.26) = 12.53, *p* < 0.001, CI: − 2.45, − 0.70, ${\eta }_{p}^{2}$= 0.03), after controlling for age, sex, education, income, habit and hours of weekly mindfulness practice, and diagnostic history of psychiatric disorder. Follow-up analyses suggested that compared to the baseline (*M*_intervention_ = 59.87, *SD* = 7.84; *M*_control_ = 59.28, *SD* = 7.94), participants from the intervention group reported significantly greater mindfulness than did those from the control group at post-intervention (*t*(202) = 3.97, *p* < 0.001, Cohen’s *d* = 0.56; *M*_intervention_ = 64.17, *SD* = 7.92; *M*_control_ = 59.81, *SD* = 7.77) and at follow-up (*t*(169) = 2.67, *p* = 0.008, Cohen’s *d* = 0.41; *M*_intervention_ = 64.73, *SD* = 7.83; *M*_control_ = 61.33, *SD* = 8.55). Within-group analyses indicated that compared to the baseline, participants from the intervention group had a significant increase of mindfulness at post-intervention (*t*(95) =  − 7.26, *p* < 0.001, Cohen’s *d* =  − 0.74) and at follow-up (*t*(72) =  − 6.20, *p* < 0.001, Cohen’s *d* =  − 0.73). Participants from the control group did not have a significant increase in mindfulness at post-intervention (*p* > 0.05), but they had a significant increase at follow-up (*t*(97) =  − 2.30, *p* < 0.001, Cohen’s *d* =  − 0.25), compared to baseline. See Fig. 4 for details.

LMM revealed non-significant group × time effects on parents’ depressive symptoms (*F*(1, 421.75) = 1.07, *p* = 0.30, CI: − 0.31, 1.00, *η*_*p*_^2^ = 0.002) and parental stress (*F*(1, 396.37) = 2.86, *p* = 0.09, CI: − 0.16, 2.16, ${\eta }_{p}^{2}$= 0.007), after controlling for the effects of sex, age, education, income, habit of mindfulness practice, hours of weekly mindfulness practice, and diagnostic history of psychiatric disorder. As such, the intervention group did not differ from the waitlist control group in changing participants’ depressive symptoms and parental stress.

## Discussion

Drawing from previous research (e.g., Williams & Penman, 2011), a 21-day mindfulness-based intervention entitled NL-MBP was developed for parents in Hong Kong and its initial efficacy was evaluated. Through a non-clinical parent sample, the brief asynchronous intervention showed changes in some areas (i.e., mindfulness, subjective well-being, and anxiety) and not others (i.e., parenting stress and depressive symptoms), after controlling the effects of sex, age, education, income, habit and hours of weekly mindfulness practice, and diagnostic history of psychiatric disorder. Initial data also suggested that the NL-MBP was acceptable and practical.

Consistent with previous studies showing the mental health benefits of mindfulness-based interventions (Bazzano et al., 2015; Benn et al., 2012; Lunsky et al., 2021), the present study indicated that the NL-MBP improved parents’ mindfulness and subjective well-being and reduced their anxiety symptoms. As discussed earlier, parenthood can be stressful, especially in a fast-paced city such as Hong Kong, whereby parents typically work long hours to afford the living expenses (Aryee et al., 1999a; Jung & Kim, 2021). Together with work and economic stress, childcare responsibilities and potential work-family conflict may further undermine parents’ well-being (Aryee et al., 1999b; Brooks-Gunn et al., 2010; Chatterji et al., 2013; Jung & Kim, 2021). Mindfulness provides an avenue to help parents respond to their everyday responsibilities. By pausing and disengaging themselves from autopilot, parents are more likely to broaden their awareness to previously unattended details, such that they could develop better subjective well-being (Garland et al., 2015). They are also less likely to chronically engage in fight or flight response, which is linked to anxiety problems (Kunimatsu & Marsee, 2012; Wheatley, 1997). For example, in the midst of a conflict, parents are more likely to pause and be aware of their physical sensations (e.g., sweaty palms), emotions (e.g., anger), and potential assumptions and biases (e.g., “my son fails the exam because he is not working hard enough”). They are also more likely to listen to themselves and other family members with full attention and respond skillfully. By pausing, attending, and skillfully responding to themselves and to others, parents are more likely to have a greater well-being.

The NL-MBP did not reduce parents’ depressive symptoms at post-intervention and the delayed follow-up. Compared to other 8-week interventions such as MBCT (Kabat-Zinn, 2009) or MBSR (Segal et al., 2018), the brevity of the present intervention, its asynchronous nature, and the lack of contact with a responsive teacher and peers might have contributed to the null findings. In addition, the baseline level of depressive symptoms was low in the present sample (*M*_intervention_ = 7.51, *SD*_intervention_ = 4.55; *M*_control_ = 8.92, *SD*_control_ = 5.43; range: 0–27), so there might have been little room for improvement. Similarly, the intervention did not reduce parental stress and contradicted previous findings (e.g., Burgdorf et al., 2019). In the present study, the mindfulness readings and practice recordings were targeted to improve parents’ well-being, rather than to reduce parental stress or enhance parent–child relationship. As a result, the null findings might have been due to the nature of the intervention. In addition, given the data were collected during the fluctuating pandemic, external factors such as COVID-19-related stress, lockdown measures, and coparenting support might also have moderated the effect of the intervention on parents’ perceived stress and burnout (e.g., Bastiaansen et al., 2021; Vaydich & Cheung, 2022).

### Limitations and Future Directions

The present findings must be interpreted in light of several limitations. First, we primarily examined one area of feasibility, i.e., initial efficacy. Although we had some data to indicate practicality and an excellent acceptability of the 21-day intervention, less than half of the participants from the intervention group responded to these items, as the items were not presented in a forced-choice response format. Future studies should ensure all participants respond to items of practicality and acceptability. Other aspects of feasibility, including demand, implementation, adaptation, integration, and expansion (Bowen et al., 2009), may also be examined through quantitative and qualitative approaches to ensure that the intervention is feasible. Relatedly, in developing the NL-MBP, we did not seek parents’ input on its content, length, and format. As parents’ comments and suggestions are vital to improving feasibility, follow-up studies could incorporate parents’ feedback on the intervention. Also, despite the advantages of the asynchronous NL-MBP, it should be noted that the intervention was conducted with a heavy reliance on self-learning, in addition to the absence of teacher-guided inquiries, peer interactions, and a responsive teacher (Cavanagh et al., 2014). Next, we only collected post-intervention data twice, i.e., immediately and 30 days after the 21-day intervention. Future research may include additional follow-up assessments to investigate the long-term benefits of NL-MBP. Importantly, more work is needed to explore the benefits of sustained practice as a result of NL-MBP.

Past research suggested that variables such as risk status, cultural values, and length of intervention may alter the effect of mindfulness on psychological functioning (Carmody & Baer, 2009; Chen & Cheung, 2021). For instance, parents high in independent self-construal (i.e., a cultural value) may prioritize individual well-being over social concerns or harmony. Such an emphasis may moderate the effect of mindfulness practice on well-being. Hence, greater attention should be paid to the role of potential moderators on parental well-being. Also, even though the NL-MBP was not designed in response to the fluctuating COVID-19 pandemic, the study was conducted during this period (i.e., between May 2020 and January 2021). To prevent the spread of COVID-19, the Hong Kong government implemented social restrictions such as school closure and “no dine-in” measures (e.g., The Standard, 2022). In the face of social restrictions, disrupted child and family routines, reduced work-life balance, reduced family support (e.g., babysitters), scarcity of sanitizing products, unemployment, and financial burden (e.g., Freisthler et al., 2021; Yuen et al., 2020), local parents were particularly vulnerable to stress, resulting in worse mental health (Chan, 2022). Therefore, replication studies are needed to examine the effects of NL-MBP when most parents are no longer experiencing the stress brought by the pandemic. Next, the present sample consisted primarily of mothers. To increase generalizability to mothers, fathers, and other caregivers, future studies may recruit a gender-balanced and diverse sample. Furthermore, we assessed the variables using self-report questionnaires, resulting in common method bias (Podsakoff et al., 2012). Method biases are major sources of measurement error, which could threaten the validity of the conclusions about the relationships between measures (Podsakoff et al., 2003). Future research may include different scale formats (e.g., Likert scales and Guttman scales), multiple reporters, and additional measures, such as physiological and observational assessments, to minimize biases.

Because this study focused on intervening at the individual parent level, the effectiveness of the intervention may be investigated in conjunction with parallel mindfulness intervention for their children. For instance, concomitant parent and child mindfulness training has shown positive outcomes for both children with ADHD and their parents (van der Oord et al., 2012). Children receiving their version of mindfulness intervention may experience better emotion regulation and reduction in behavioral problems (Meiklejohn et al., 2012), thereby creating more mental space for their parents to benefit from the parent mindfulness intervention. Experiencing similar intervention programs over the same period may also facilitate parent–child bonding as they share common conversation and goal and reinforce each other’s gains.

## Data Availability

The dataset analyzed in this article is not publicly available. Requests to access the dataset should be directed to Rebecca Y. M. Cheung, Ph.D., School of Psychology and Clinical Language Sciences, Harry Pitt Building, University of Reading, Earley Gate, Reading, RG6 6ES, e-mail: rebecca.cheung@reading.ac.uk.
